# A Review on the Phytochemistry, Pharmacology, and Pharmacokinetics of Amentoflavone, a Naturally-Occurring Biflavonoid

**DOI:** 10.3390/molecules22020299

**Published:** 2017-02-16

**Authors:** Sheng Yu, Hui Yan, Li Zhang, Mingqiu Shan, Peidong Chen, Anwei Ding, Sam Fong Yau Li

**Affiliations:** 1 Jiangsu Collaborative Innovation Center of Chinese Medicinal Resources Industrialization, College of Pharmacy, Nanjing University of Chinese Medicine, 138 Xianlin Road, Nanjing 210023, China; yusheng1219@163.com (S.Y.); glory-yan@163.com (H.Y.); zhangliguanxiong@163.com (L.Z.); chenpeidong1970@163.com (P.C.); awding105@163.com (A.D.); 2 Department of Chemistry, National University of Singapore, Singapore 117543, Singapore; chmlifys@nus.edu.sg

**Keywords:** amentoflavone, biflavonoid, natural derivatives, pharmacokinetics, pharmacology, phytochemistry

## Abstract

Amentoflavone (C_30_H_18_O_10_) is a well-known biflavonoid occurring in many natural plants. This polyphenolic compound has been discovered to have some important bioactivities, including anti-inflammation, anti-oxidation, anti-diabetes, and anti-senescence effects on many important reactions in the cardiovascular and central nervous system, etc. Over 120 plants have been found to contain this bioactive component, such as Selaginellaceae, Cupressaceae, Euphorbiaceae, Podocarpaceae, and Calophyllaceae plant families. This review paper aims to profile amentoflavone on its plant sources, natural derivatives, pharmacology, and pharmacokinetics, and to highlight some existing issues and perspectives in the future.

## 1. Introduction

**Figure 1:**
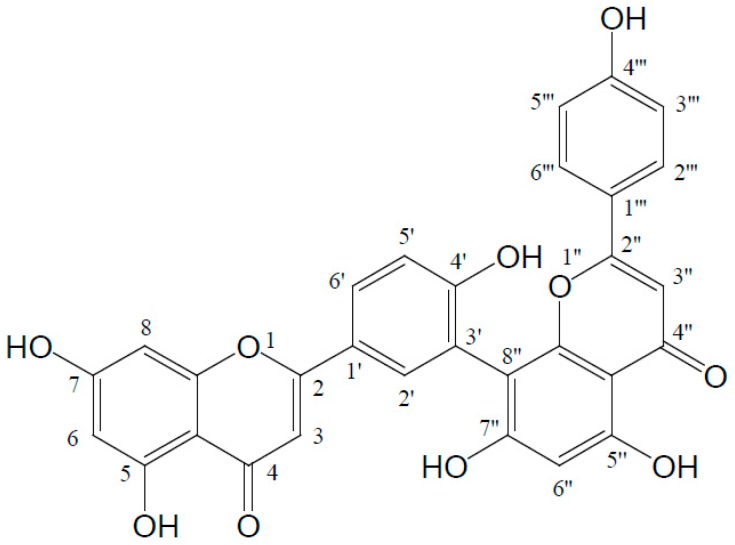
Chemical structure of amentoflavone.

Amentoflavone (C_30_H_18_O_10_) is a common biflavonoid chemically named as 8-[5-(5,7-dihydroxy-4-oxo-4*H*-chromen-2-yl)-2-hydroxyphenyl]-5,7-dihydroxy-2-(4-hydroxyphenyl)-4*H*-chromen-4-one, which naturally occurs in many plants. It is also considered as an apigenin dimer linked by a C3′-C8′′ covalent bond (Figure 1). This compound was firstly isolated by Okigawa and his colleagues in 1971 from three plants of the *Selaginella* species (*Selaginella tamariscina* (Beauv.) Spring, *Selaginella nipponica*, and *Selaginella pachystachys*) [1]. From then on, phytochemical researchers have isolated and identified this biflavonoid from more than 120 plants, some of which have been used as traditional folk medicines in many regions of the world for even thousands of years. With the development of modern pharmacology, more and more evidence has proved many of the bioactivities of amentoflavone, including anti-oxidant [2], anti-inflammatory [3], anti-senescence [4], anti-tumor [5], anti-virus [6], and anti-fungal [7] effects, as well as therapeutic effects on the central nervous system [8] and cardiovascular system [9], etc. With its good pharmacological performance and high content, amentoflavone is even listed as the chemical marker of Selaginellae Herba (“Juanbai” in Chinese, which represents the whole plants of *Selagenella tamariscina* or *Selaginella pulvinata*) for quality evaluation in the *Chinese Pharmacopoeia* [10].

Due to its large range of bioactivities and originating from nature, amentoflavone has attracted increasing focus from a number of research fields. Here, in this paper, we aim to provide a review of this naturally-occurring biflavonoid, describing its sources, natural derivatives, pharmacological effects, and pharmacokinetics, and to help researchers understand and utilize it in a better way.

## 2. Sources

As a polyphenolic compound, amentoflavone exists in a large number of plants (Table 1). To our knowledge, the major sources are the plants of Calophyllaceae, Clusiaceae, Cupressaceae, Euphorbiaceae, and Selaginellaceae families, and *Calophyllum*, *Garcinia*, and *Selaginella* species, etc. Some of these plants have been used as folk phytomedicines for a very long time, such as *Gingko biloba*, *Lobelia chinensis*, *Polygala sibirica*, *Ranunculus ternatus*, *Selaginella pulvinata*, *Selagenella tamariscina* for traditional Chinese medicines (TCMs), *Calophyllum inophyllum*, *Selaginella bryopteris* for traditional Indian medicines, *Byrsonima intermedia* for traditional American medicine, and *Cnestis ferruginea* and *Drypetes gerrardii* for traditional African medicines.

## 3. Extraction and Isolation

To obtain amentoflavone from plants as much as possible, and to fully utilize these plant sources, some studies have been carried out to optimize the extraction technology. A central composite design (CCD) method was used to optimize the extraction technology of amentoflvone from *Taxus chinensis* by supercritical-CO_2_ fluid extraction (SFE-CO_2_) with methanol as a co-solvent. The highest yield reached 4.47 mg/g when the plant was extracted with 78.5% ethanol at 48 °C under a pressure of 25 Mpa for 2.02 h [135]. With 35% water in ChCl/1,4-butanediol (1:5) as the extraction solvent, 0.518 mg/g of amentoflavone could be extracted from *Chamaecyparis obtusa* leaves at 70 °C for 40 min with a solid/liquid ratio of 0.1 g/mL, which was optimized by a response surface methodology [136].

Like other phytochemicals, separation and isolation of amentoflavone were mainly performed with conventional thin layer chromatography [23,24] and column chromatography, in which silica gel [15,18,25], polyamide [16], macroporous adsorption resin [85,86], octadecyl silane [11,22], middle chromatogram isolation (MCI) gel [51], and gel (Sephadex LH-20) [12,13,27] were used as stationery phases. In most cases, some of the above methods were combined for use [51,63,82,88,115,137]. Additionally, as a novel isolation method, high-speed counter-current chromatography (HSCCC) has been widely used to isolate this bioflavonoid. A preparative isolation method with HSCCC was adopted to isolate amentoflavone from *Selaginella doederleinii*. The mixed solvent consisting of *n*-hexane:ethyl acetate:methanol:water (1:2:1.5:1.5, *v*/*v*/*v*/*v*) was employed for HSGCC of ethyl acetate extract of this plant. As a result, with an approximate yield of 0.34 mg from 1 g of crude plant, amentoflavone of 91.4% purity was obtained [138]. In another experiment, with HSCCC and *n*-hexane:ethyl acetate:methanol:water (2.2:2.8:2:3, *v*/*v*/*v*), 65.31 mg amentoflavone (98% purity) was isolated from approximately 2.5 g of *Selaginella tamariscina* [139].

## 4. Natural Derivatives

**Figure 2:**
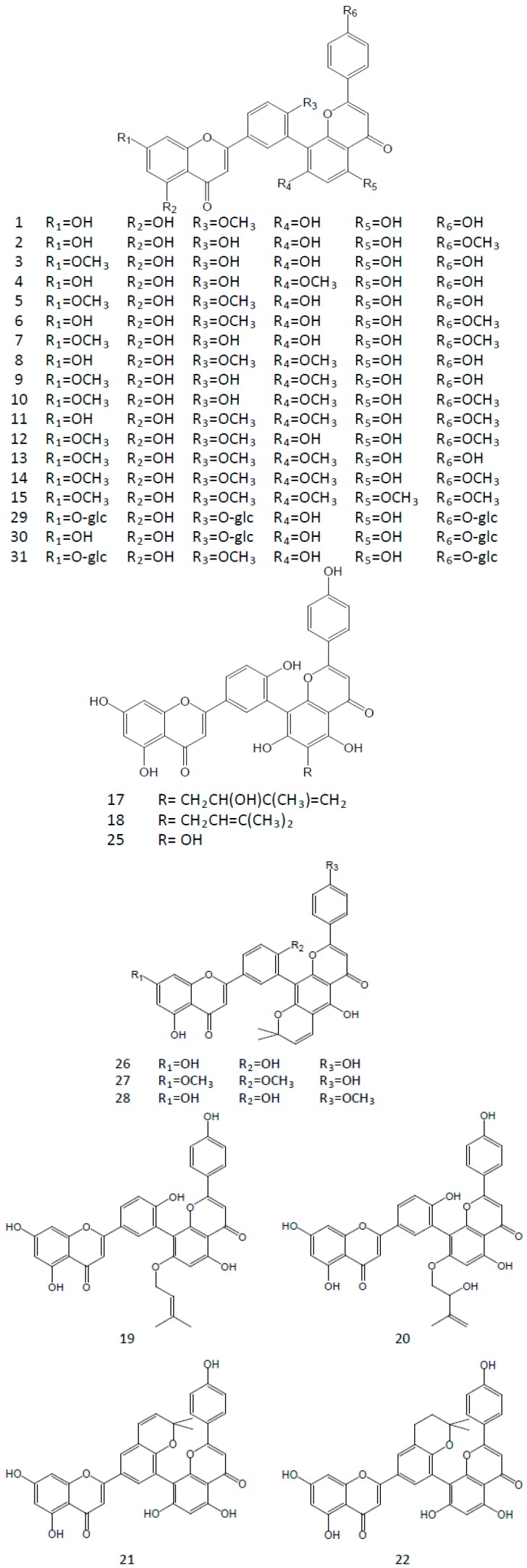
Chemical structures of natural derivatives of amentoflavone in plants.

There are also a large number of derivatives with different substitution positions and types in the natural plants (Figure 2). In most cases, they exist in the same plant with amentoflavone.

Amentoflavone is considered as a dimer of two apigenins with six hydroxyl groups on the positions of C5, C7, C4′, C5′′, C7′′, and C4′′′ in its structure (Figure 1). Among these groups the C7-, C4′-, C7′′-, or C4′′′-hydroxyl group is easily substituted by a methoxyl group. 7-*O*-methylamentoflavone (sequoiaflavone), 4′-*O*-methylamentoflavone (bilobetin), 7′′-*O*-methylamentoflavone (sotetsuflavone), and 4′′′-*O*-methylamentoflavone (podocarpusflavone A) are the natural derivatives with a single methoxyl group. There are five derivatives with two methoxyl groups isolated in the plants, i.e., 7,4′′′-di-*O*-methylamentoflavone (podocarpusflavone B), 4′,4′′′-di-*O*-methylamentoflavone (isoginkgetin), 7,4′-di-*O*-methylamentoflavone (ginkgetin), 7,7′′-di-*O*-methylamentoflavone, and 4′,7′′-tri-*O*-methylamentoflavone. 7,4′,7′′-tri-*O*-methylamentoflavone, 7,4′,4′′′-tri-*O*-methylamentoflavone (sciadopitysin), 7,7′′,4′′′-tri-*O*-methylamentoflavone (heveaflavone), and 4′,7′′,4′′′-tri-*O*-methylamentoflavone (kayaflavone) are the derivatives with three methoxyl groups. Furthermore, 7,4′,7′′,4′′′-tetra-*O*-methylamentoflavone has also been found in some plants. Additionally, there are some other derivatives, such as 6-methy-7,4′-di-*O*-methylamentoflavone (taiwanhomoflavone A), 6′′-*O*-hydroxyamentoflavone (sumaflavone), 3′′′-*O*-methylamentoflavone, 5′-hydroxyamentoflavone, and some glycosides. All of the compounds above and their plant sources are listed in Table 2.

In the structure of amentoflavone, carbon-carbon double bonds of C2-C3 and C2′′-C3′′ are easily hydrogenated, too. In a large number of plants, the hydrogenation products present include (2*S*)-2,3-dihydroamentoflavone, (2′′*S*)-2′′,3′′-dihydroamentoflavone, and (2*S*,2′′*S*)-2,3,2′′,3′′-tetrahydroamentoflavone, along with their C4′-*O*-methyl derivatives, such as (2*S*)-2,3-dihydro-4′-*O*-methylamentoflavone, (2′′*S*)-2′′,3′′-dihydro-4′-*O*-methylamentoflavone, (2*S*,2′′*S*)-2,3,2′′,3′′-tetrahydro-4′-*O*-methylamentoflavone, and their glycosides (Table 3).

## 5. Pharmacology

As a ubiquitous biflavonoid, amentoflavone has been found with a large number of pharmacological functions, such as anti-inflammation, anti-oxidation, anti-tumor, anti-senescence, anti-virus, anti-diabetes, neuroprotective activities, and effects on cardiovascular system and central nervous system.

### 5.1. Anti-Inflammation and Anti-Oxidation

Oxidative stress response is one part of inflammatory response. Amentoflavone, isolated from *Garcinia brasiliensis*, exhibited inhibitory effects on the productions of superoxide anion and total reactive oxygen species (ROS) inphorbol 12-myristate 13-acetate-stimulated human neutrophils. In human erythrocytes induced by 2,2′-azobis(2-amidinopropane) hydrochloride, it also inhibited the oxidant hemolysis and lipid peroxidation [2].

In rat astrocytoma cell line, lipopolysaccharide (LPS) could increase NO, ROS, malondialdehyde (MDA), and decrease reduced-glutathione (GSH), while tumor necrosis factor-α (TNF-α) was increased by LPS in a human monocytic leukemia cell line. All of the changes above were attenuated by amentoflavone significantly. However, there were no notable effects on the cells [164]. In RAW 264.7 cells stimulated with LPS, amentoflavone was observed to suppress the production of NO, prostaglandin E-2 (PGE-2), and the nuclear translocation of c-Fos, a subunit of activator protein (AP)-1. Additionally, extracellular signal-regulated kinase (ERK), which mediated c-Fos translocation, was inhibited by the active biflavonoid [165]. In the supernatant media of human peripheral blood mononuclear cells (PBMCs), amentoflavne could inhibit the increases of interleukin-1β (IL-1β), IL-6, TNF-α, and PGE2 induced by phytohaemagglutinin (PHA) [3].

The IC_50_ values of amentoflavone were 31.85 ± 4.75, 198.75 ± 33.53, 147.14 ± 20.95, 75.15 ± 10.52, 93.75 ± 16.36, 167.69 ± 13.90, and 137.95 ± 19.86 μM, respectively, for DNA, cytosine, uracil, adenine, thymine, guanine, and deoxyribose damage. Radical-scavenging assays indicated that amentoflavone could effectively scavenge center dot O^2−^, DPPH, ABTS^+^ radicals with IC_50_ values of 8.98 ± 0.23, 432.25 ± 84.05, 7.25 ± 0.35 μM, respectively [166].

### 5.2. Anti-Tumor

Amentoflavone exerted good cytotoxic effect on cervical adenocarcinoma (HeLa) cells with IC_50_ values of 20.7 μM [5].

After breast cancer MCF-7 cells were treated with amentoflavone, there were some cellular changes, including DNA and nuclear fragmentation, and down-regulation of calcium and intracellular reactive oxygen species. Additionally, some marks of mitochondrial-mediated apoptosis were observed, such as the activation of caspase 3, the reduction of mitochondrial inner-membrane potential, and the release of cytochrome c from mitochondria [167].

Amentoflavone also could significantly inhibit solid tumor development that was induced by B16F-10 melanoma in C57BL/6 mice. The mechanism might be related to inhibiting cell progression from G0/G1 to S phase and to regulating genes which were involved in cell cycle and apoptosis, such as P21, P27, Bax, caspase-9, etc. [168].

Recently, fatty acid synthase (FASN) has been considered as a potential target to treat cancer. Some studies indicated that amentoflavone could inhibit FASN expression in human epidermal growth factor receptor 2 (HER2)-positive human breast carcinoma SKBR3 cells. The inhibition decreased the translocation of sterol regulatory element-binding protein 1 (SREBP-1) in SKBR3 cells. The biflavonoid was also found to down-regulate HER2 protein and mRNA, to up-regulate polyoma enhancer activator 3 (PEA3), a transcriptional repressor of HER2 and to inhibit phosphorylation of protein kinase B (PKB), mechanistic target of rapamycin (mTOR) and c-Jun N-terminal kinases (c-JNK) [169]. In another experiment, amentoflavone was observed to increase the cleavage-activity of caspase-3, to suppress SKBR3 cell activity, and to have no effect on FASN-nonexpressed NIH-3T3 normal cell growth [170].

### 5.3. Anti-Senescence

Ultraviolet B (UVB) irradiation was found to increase the levels of Lamin A and phospho-H2AX protein in normal human fibroblasts. These cases were present in premature aging diseases or normally old individuals. An investigation indicated amentoflavone was able to ameliorate these damages and to protect nuclear aberration significantly, which showed the anti-senescence activity for some skin aging processes related with UVB [4]. Another investigation in UVB-induced normal human fibroblasts found that amentoflavone could inhibit the activation of ERK without affecting ERK protein level, p38, and JNK activation. In addition, the biflavonoid could decrease phospho-c-Jun and c-Fos protein expressions, which were AP-1 transcription factor components. The findings suggested the potential of amentoflavone to prevent or treat skin photoaging [171].

### 5.4. Anti-Diabetes

Amentoflavone was observed to ameliorate glucose disorder, regulate insulin secretion, and restore the pancreas in streptozotocin-induced diabetic mice and the optimum dose was 60 mg/kg [172]. In another anti-diabetes study, this active biflavonoid showed its activities against α-glucosidase (IC_50_ 8.09 ± 0.023 μM) and α-amylase (IC_50_ 73.6 ± 0.48 μM) [58].

Inhibition of protein tyrosine phosphatase 1B (PTP1B) has been considered as a strategy to treat type 2 diabetes. Amentoflavone was screened to inhibit PTP1B with IC_50_ value of 7.3 ± 0.5 μM and proved to be a non-competitive inhibitor of PTP1B by kinetic study. There was a dose-dependent increase in tyrosine phosphorylation of insulin receptor (IR) after 32D cells with overexpression of IR were treated with amentoflavone [173].

### 5.5. Anti-Virus

Amentoflavone exhibited its anti-dengue potential in a screening experiment, which may be mediated by inhibiting Dengue virus NS5 RNA-dependent RNA polymerase [6]. Among the isolated twelve components from *Torreya nucifera* with a bioactivity guide, amentoflavone was proved as the most active one to inhibit severe acute respiratory syndrome coronavirus (SARS-CoA) with IC_50_ value of 8.3 μM. The effect was concluded relative to the inhibition of chymotrypsin-like protease (3CL^pro^) [131]. Amentoflavone was also found to decrease Coxsackievirus B3 (CVB3) replication by inhibiting fatty acid synthase (FAS) expression [174]. Moreover, in cases of human immunodeficiency virus (HIV) and respiratory syncytial virus (RSV), amentoflavone showed good performance with IC_50_ values of 119 µM [102] and 5.5 μg/mL [120], respectively.

### 5.6. Effects on Central Nervous System

After amentoflavone was isolated from *Cnestis ferruginea*, Ishola et al. carried out some investigations about its effects on central nervous system. In one pharmacological investigation, oral administration of amentoflavone was proved to attenuate depression induced by metergoline (5-HT2 receptor antagonist), prazosin (α1-adrenoceptor antagonist), or yohimbine (α2-adrenoceptor antagonist), and to ameliorate anxiety stimulated by flumazenil (ionotropic GABA receptor antagonist). These findings suggested that the active biflavonoid showed the antidepressant and anxiolytic effects through interactions with the receptors above [175]. In another study, it was found that the naturally-occurring biflavonoid could prevent scopolamine-induced memory impairment, inhibit AChE and enhance antioxidant enzyme activity in mice, which exhibited its protection against memory deficits [176].

In glutamate injured HT22 hippocampal cells, amentoflavone showed neuroprotective activity. The active compound was able to restore the reduced superoxide dismutase (SOD) activity, glutathione reductase (GR) activity and glutathione content induced by glutamate. Additionally, it was found to prevent the phosphorylation of ERK1/2 [177]. Amentoflavone also exerted neuroprotective activity in pilocarpine-induced epileptic mice. After preventive administration of the biflavonoid for three consecutive days, the model mice showed some signs of improvement, including reduction of epileptic seizures, shortened attack time, reduction in hippocampal neuron loss and apoptosis, and suppressed nuclear factor-kappa B (NF-κB) activation and expression [8].

### 5.7. Effects on the Cardiovascular System

Amentoflavone was tested to have a vasorelaxant effect on thoracic aortic blood vessels of rats in vitro, which was concluded as being endothelium-dependent and involved with NO [178].

Amentoflavone also had a protective effect on vascular endothelial cells. The viability of human umbilical vein endothelial cells (HUVECs) was promoted and the ratio of cells at S phase was increased by treatment with this biflavonoid [179]. Some results of cell studes indicated that amentoflavone could increase the NO content, decrease the levels of VCAM-1, E-selectin, IL-6, IL-8, and ET-1, enhance SOD activity, reduce MDA content, downregulate the protein expressions of VCAM-1, E-selectin, and NF-κB p65, up-regulate IκBα, and attenuate the NF-κB p65 transfer to the cell nucleus, which proved its protection on vascular endothelial cells [9].

Cyclic adenosine monophosphate (cAMP) phosphodiesterase (PDE) inhibitor has been found to inhibit the activity of cAMP-PDE-3 in myocardial cells and vascular smooth muscle cells, which could enhance myocardial contraction, expand peripheral vessels, and improve hemodynamics of heart failure patients. Amentoflavone showed a potent inhibitory function on cAMP-PDE [180]. The effect study of amentoflavone on isolated rat heart exhibited that the phytochemical significantly increased the beat rate at dosage of 10–50 μg/mL [181].

### 5.8. Antifungal Activity

Amentoflavone was investigated to have antifungal activity against several pathogenic fungal strains, including *Candida albicans*, *Saccharomyces cerevisiae*, and *Trichosporon beigelii*. In *Candida albicans*, it could stimulate the intracellular trehalose accumulation and disrupt the dimorphic transition, which meant a stress response to the component [182]. Further research on its antifungal mechanism of *Candida albicans* suggested that this active phytochemical arrested cell cycles during the S-phase and inhibited cell proliferation and division [183]. The anti-candida activity was proved to be related to apoptotic cell death, which may be associated with the mitochondrial dysfunction. Additionally, hydroxyl radicals induced by amentoflavone may play a significant role in apoptosis [7].

### 5.9. Other Bioactivities

In addition to the pharmacological functions above, significant evidence showed its other bioactivities (Table 4), such as anti-hyperlipidemia [184], anti-hypertrophic scar [185], anti-psoriasis [186], anti-ulcerative colitis [187], hepatoprotection [184], osteogenesis effect [188] and radioprotection [189].

## 6. Pharmacokinetics

In recent years, pharmacokinetic studies of extracts and bioactive compounds from traditional Chinese medicine and natural medicine have become research highlights. As a representative biflavonoid with several pharmacological functions, amentoflavone was not an exception.

In a pharmacokinetic investigation, amentoflavone was administrated to rats with different types including oral gavage (*po*, 300 mg/kg), intravenous (*iv*, 10 mg/kg) and intraperitoneal (*ip*, 10 mg/kg) injection. As a result, 90.7% of the total amentoflavone was discovered to circulate as conjugated metabolites after *po* administration. In the plasma of rats with *iv* and *ip* injection, 73.2% ± 6.29% and 70.2% ± 5.18% of the total amentoflavone was present as conjugated metabolites. In addition, the bioavailability of this compound with *po* administration was 0.04% ± 0.01%, much lower than that with *ip* injection (77.4% ± 28.0%) [190].

Pharmacokinetic characteristic of amentoflavone individually or together with other components in normal rats and hyperlipidemic model rats have been studied and compared [191]. In the case of oral administration of only this biflavonoid, T_1/2_ and T_max_ of amentoflavone were determined as 2.06 h ± 0.13 h, 1.13 h ± 0.44 h in normal rats and 1.91 h ± 0.32 h, 0.96 h ± 0.10 h in model rats, respectively. Shixiao San is a famous TCM formula containing amentoflavone [192]. After oral administration of a Shixiao San decotion, T_1/2_ and T_max_ of amentoflavone were determined as 3.34 h ± 0.37 h, 4.00 h ± 0.00 h in normal rats, and 4.19 h ± 0.64 h, 4.17 h ± 0.40 h in model rats.

## 7. Conclusions and Future Perspectives

From the contents above, we could conclude that amentoflavone is a bioactive biflavonoid with a variety of pharmacological effects, which has been derived from many natural plants.

Emerging pharmacological evidence has proved the effects of amentoflavone on various aspects, including anti-inflammation, anti-oxidation, anti-diabetes, anti-senescence, anti-virus, anti-tumor activities, and effects on the central nervous system and cardiovascular system. However, the majority of these bioactivity data came from studies involved with cells in vitro, while the number of studies with model animals in vivo was very low. As we know, bioactivity in vitro is unable to represent and explain biological effect in vivo, while pharmacological investigations in model animals are indispensable prior to clinical use. Thus, some bioactivities in vitro should be confirmed and proved by integral animal experiments in the future. In terms of present pharmacokinetic study, the findings have suggested that amentoflavone metabolism procedure was very rapid and there was also a very low bioavailability after oral administration of this biflavonoid in rats. This may be one reason why fewer animal model experiments have been performed. We speculate that improving the bioavailability with introduction of structural modification, precursor synthesis, or particular pharmaceutical necessities may be one focus of amentoflavone studies. Meanwhile, since there are some differences of pharmacokinetics between normal and model animals, concerning the specific pharmacological effects, the pharmacokinetic investigations on corresponding model animals should also be carried out.

Amentoflavone has been found, isolated, and identified in over 120 natural plants, which exhibited its rich plant source. The content of any phytochemical varies very much in different species or in different regions. Among 11 plants from *Selaginella* species, the biflavonoid was found with the high contents between 1.0% and 1.1% in *Selaginella sinensis*, *Selaginella davidii*, and *Selaginella mollendorfii* from some specific production areas, while the contents were no more than 1.0% in the rest, and even below 0.1% in some [193,194]. It is well-known that extraction yield will be lower than the determined content. In addition, most of the sources are perennial plants and their recovery or reproduction will last not a short time. Thus, at present, plant-derived preparation seems to cost too much. This may be another reason of fewer animal model experiments, which would need much higher amounts of the biflavonoid than cell experiments. We must find some solutions to get the sufficient quantity for studies in the future, such as looking for other plants with much higher contents, biological synthesis, and even chemical synthesis.

Taken together, since amentoflavone is a promising and naturally-occurring biflavonoid with so many bioactivities, its systematic druggability research as a candidate drug is obviously necessary, including its preparation study (extraction and isolation from plants, chemical synthesis, or biological synthesis), structural modification study, Absorption-Distribution-Metabolism-Excretion (ADME) study in normal animals and animal models, acute and chronic toxicological studies. Thus, we can make full use of amentoflavone as a drug and employ it in the prevention and treatment of diseases.

In summary, this paper has provided a full-scale profile of amentoflavone on its plant sources, natural derivatives, pharmacology, and pharmacokinetics, and also proposed some issues and perspectives which may be of concern in the future. We believe this literature review will help us more comprehensively understand, and take advantage more fully, the naturally-occurring biflavonoid amentoflavone.

## Figures and Tables

**Table 1:** Plants containing amentoflavone.

No.	Plant	Family	Part	References
1	*Amanoa almerindae*	Phyllanthaceae	aerial parts	[11]
2	*Alchornea glandulosa*	Euphorbiaceae	leaves	[12]
3	*Alchornea triplinervia*	Euphorbiaceae	leaves	[13]
4	*Aletris spicata*	Liliaceae	herbs	[14]
5	*Allanblackia monticola*	Guttiferae	leaves	[15]
6	*Androsace umbellata*	Primulaceae	whole plants	[16]
7	*Antidesma bunius*	Phyllanthaceae	leaves	[17]
8	*Antidesma laciniatum*	Euphorbiaceae	leaves	[18]
9	*Biophytum sensitivum*	Oxalidaceae	roots	[19]
10	*Biota semipervirens*	Cupressaceae	leaves	[20]
11	*Byrsonima crassa*	Malpighiaceae	leaves	[21]
12	*Byrsonima intermedia*	Malpighiaceae	leaves	[22]
13	*Caesalpinia pyramidalis*	Leguminosae	leaves	[23]
14	*Callitris rhomboidea*	Cupressaceae	leaves	[24]
15	*Calocedrus microlepic* var. *formosana formosana*	Cupressaceae	leaves	[25]
16	*Calophyllum brasiliense*	Calophyllaceae	leaves	[26]
17	*Calophyllum ferrugineum*	Calophyllaceae	barks, leaves	[27]
18	*Calophyllum flavoramulum*	Calophyllaceae	leaves	[28]
19	*Calophyllum incrassatum*	Calophyllaceae	barks, leaves	[29]
20	*Calophyllum inophylloide*	Calophyllaceae	heartwood	[30]
21	*Calophyllum inophyllum*	Calophyllaceae	leaves	[31]
22	*Calophyllum membranaceum*	Guttiferae	roots	[32]
			leaves	[33]
23	*Calophyllum pinetorum*	Guttiferae	stem barks, leaves	[34]
24	*Calophyllum rivulare*	Calophyllaceae	leaves	[35]
25	*Calophyllum symingtonianum*	Calophyllaceae	barks, leaves	[36]
26	*Calophyllum venulosum*	Calophyllaceae	leaves	[37]
27	*Campylospermum calanthum*	Ochnaceae	leaves	[38]
28	*Campylospermum mannii*	Ochnaceae	leaves	[39]
29	*Canarium album*	Burseraceae	fruits	[40]
			leaves	[41]
30	*Canarium pimela*	Burseraceae	fruits	[42]
31	*Canarium schweinfurthii*	Burseraceae	seeds	[43]
32	*Casearia clarkei*	Flacourtiaceae	leaves	[44]
33	*Celaenodendron mexicanum*	Euphorbiaceae	leaves, twigs	[45]
34	*Cephalotaxus fortunei*	Cephalotaxaceae	leaves	[46]
35	*Cephalotaxus koreana*	Cephalotaxaceae	leaves, twigs	[47]
36	*Cephalotaxus oliveri*	Cephalotaxaceae	leaves	[48]
37	*Chamaecyparis obtusa*	Cupressaceae	leaves	[49]
38	*Chrozophora tinctoria*	Euphorbiaceae	aerial parts	[3]
39	*Cnestis ferruginea*	Connaraceae	roots	[50]
40	*Cunninghamia lanceolata*	Taxodiaceae	branches, leaves	[51]
41	*Cupressocyparis leylandii*	Cupressaceae	leaves	[52]
42	*Cupressus chengiana*	Cupressaceae	-	[53]
43	*Cupressus sempervirens*	Cupressaceae	leaves	[54]
44	*Cycas beddomei*	Cycadaceae	cones	[55]
45	*Cycas circinalis*	Cycadaceae	leaflets	[56]
46	*Cycas panzhihuaensis*	Cycadaceae	flowers	[57]
47	*Cycas pectinata*	Cycadaceae	fruits	[58]
48	*Cycas revoluta*	Cycadaceae	leaflets	[56]
49	*Dacrydium araucarioides*	Podocarpaceae	leaves	[6]
50	*Decussocarpus rospigliosii*	Podocarpaceae	leaves	[59]
51	*Discocleidion rufescens*	Euphorbiaceae	aerial parts	[60]
52	*Dorstenia barteri*	Moraceae	twigs	[61]
53	*Drypetes gerrardii*	Euphorbiaceae	stems	[62]
54	*Drypetes hainanensis*	Euphorbiaceae	leaves, stems	[63]
55	*Elateriospermum tapos*	Euphorbiaceae	stems, leaves	[64]
56	*Galeobdolon chinense*	Labiatae	whole plants	[65]
57	*Garcinia bakeriana*	Clusiaceae	leaves	[66]
58	*Garcinia brasiliensis*	Clusiaceae	branches, leaves	[2]
59	*Garcinia brevipedicellata*	Clusiaceae	stem heartwood	[67]
60	*Garcinia cowa*	Clusiaceae	fruits	[68]
61	*Garcinia intermedia*	Clusiaceae	leaves	[69]
62	*Garcinia livingstonei*	Clusiaceae	leaves	[70]
			fruits	[71]
63	*Garcinia merguensis*	Clusiaceae	twigs	[72]
64	*Garcinia subelliptica*	Clusiaceae	leaves	[73]
65	*Garcinia xanthochymus*	Clusiaceae	fruits	[74]
66	*Gingko biloba*	Ginkgoaceae	leaves	[75]
67	*Hypericum connatum*	Hypericaceae	aerial parts	[76]
68	*Hypericum perforatum*	Hypericaceae	aerial parts	[77]
69	*Hyeronima alchorneoides*	Euphorbiaceae	leaves	[78]
70	*Juniperus occidentalis*	Cupressaceae	leaves	[79]
71	*Juniperus rigida*	Cupressaceae	leaves, twigs	[80]
72	*Lanaria lanata*	Lanariaceae	whole plants	[81]
73	*Lobelia chinensis*	Campanulaceae	whole plants	[82]
74	*Lonicera chrysantha*	Caprifoliaceae	aerial parts	[83]
75	*Lonicera macranthoides*	Caprifoliaceae	stems, leaves	[84]
76	*Lonicera similes*	Caprifoliaceae	flower buds	[85]
77	*Luxemburgia nobilis*	Ochnaceae	branches, leaves	[86]
78	*Lysimachia christinae*	Primulaceae	whole plants	[87]
79	*Mangifera indica*	Anacardiaceae	leaves	[88]
80	*Manihot esculenta*	Euphorbiaceae	stems	[89]
81	*Microbiota decussata*	Cupressaceae	leaves	[90]
82	*Nandina domestica*	Berberidaceae	fruits	[91]
83	*Nanuza plicata*	Velloziaceae	leaves	[92]
84	*Ochna schweinfurthiana*	Ochnaceae	barks	[5]
85	*Ouratea parviflora*	Ochnaceae	leaves	[93]
86	*Ouratea semiserrata*	Ochnaceae	branches, leaves	[94]
87	*Ouratea sulcata*	Ochnaceae	aerial parts	[95]
88	*Pistacia chinensis*	Anacardiaceae	buds, inflorescences	[96]
89	*Podocarpus imbricadus*	Podocarpaceae	barks, leaves	[97]
90	*Polygala sibirica*	Polygalaceae	aerial parts	[98]
91	*Ranunculus ternatus*	Ranunculaceae	root tubers	[99]
92	*Retrophyllum rospigliosii*	Podocarpaceae	leaves	[100]
93	*Rhus pyroides*	Anacardiaceae	leaves	[101]
94	*Rhus succedanea*	Anacardiaceae	leaves, twigs	[102]
95	*Sabina pingii* var. *wilsonii*	Cupressaceae	leaves, twigs	[103]
96	*Sabina sinoalpina*	Cupressaceae	-	[104]
97	*Sabina vulgaris*	Cupressaceae	leaves	[105]
98	*Selaginella bryopteris*	Selaginellaceae	whole plants	[106]
99	*Selaginella chrysocaulos*	Selaginellaceae	whole plants	[106]
100	*Selaginella delicatula*	Selaginellaceae	whole plants	[107]
101	*Selaginella denticulata*	Selaginellaceae	whole plants	[108]
102	*Selaginella doederleinii*	Selaginellaceae	whole plants	[109]
103	*Selaginella involvens*	Selaginellaceae	whole plants	[110]
104	*Selaginella labordei*	Selaginellaceae	whole plants	[111]
105	*Selaginella moellendorffii*	Selaginellaceae	whole plants	[112]
106	*Selaginella nipponica*	Selaginellaceae	leaves	[1]
107	*Selaginella nothohybrida*	Selaginellaceae	whole plants	[113]
108	*Selaginella pachystachys*	Selaginellaceae	leaves	[1]
109	*Selaginella pulvinata*	Selaginellaceae	-	[114]
110	*Selaginella remotifolia*	Selaginellaceae	-	[115]
111	*Selaginella rupestris*	Selaginellaceae	whole plants	[116]
			leaves	[117]
112	*Selaginella sanquinolenta*	Selaginellaceae	-	[118]
113	*Selaginella selaginoides*	Selaginellaceae	whole plants	[119]
114	*Selaginella sinensis*	Selaginellaceae	herbs	[120]
115	*Selaginella stauntoniana*	Selaginellaceae	whole plants	[121]
116	*Selaginella tamariscina*	Selaginellaceae	whole plants	[122]
			leaves	[1]
117	*Selaginella uncinata*	Selaginellaceae	herbs	[123]
118	*Selaginella willdenowii*	Selaginellaceae	leaves	[124]
119	*Speranskia Tuberculata*	Euphorbiaceae	aerial parts	[125]
120	*Struthiola argentea*	Thymelaeaceae	whole plants	[126]
121	*Taxus baccata*	Taxaceae	needles	[127]
122	*Thuja orientalis*	Cupressaceae	leaves	[128]
			fruits	[129]
123	*Tmesipteris tannensis*	Psilotaceae	-	[130]
124	*Torreya nucifera*	Taxaceae	leaves	[131]
125	*Torreya yunnanensis*	Taxaceae	leaves, twigs	[132]
126	*Viburnum chinshanense*	Caprifoliaceae	aerial parts	[133]
127	*Zabelia tyaihyonii*	Caprifoliaceae	leaves	[134]

-: not mentioned.

**Table 2:** Substituted derivatives of amentoflavone.

No.	Compounds	Sources
1	Bilobetin	*Celaenodendron mexicanum* [45], *Cephalotaxus koreana* [47], *Chamaecyparis obtusa* [49], *Cycas circinalis* [56], *Dacrydium araucarioides* [6], *Gingko biloba* [140], *Ranunculus ternatus* [99], *Selaginella bryopteris* [106,141], *Selaginella moellendorffii* [142,143], *Selaginella uncinata* [137], *Selaginella willdenowii* [124], *Taxus baccata* [127], *Torreya nucifera* [131]
2	Podocarpusflavone A	*Allanblackia monticola* [15], *Antidesma bunius* [17], *Caesalpinia pyramidalis* [23], *Celaenodendron mexicanum* [144], *Chamaecyparis obtusa* [49], *Cupressocyparis leylandii* [52], *Cycas panzhihuaensis* [57], *Cycas revoluta* [56], *Decussocarpus rospigliosii* [59], *Garcinia bakeriana* [66], *Garcinia brevipedicellata* [67], *Garcinia intermedia* [69], *Garcinia livingstonei* [70], *Garcinia subelliptica* [73], *Ouratea semiserrata* [94], *Podocarpus brevifolius* [145], *Ranunculus ternatus* [99], *Retrophyllum rospigliosii* [100], *Sabina pingii* var. *wilsonii* [103], *Sabina vulgaris* [105], *Selaginella moellendorffii* [112,142], *Taxus baccata* [127]
3	sequoiaflavone	*Amanoa almerindae* [11], *Amentotaxus yunnanensis* [132], *Androsace umbellata* [16], *Campylospermum calanthum* [38], *Chamaecyparis obtusa* [49], *Cupressocyparis leylandii* [52], *Dacrydium araucarioides* [6], *Decussocarpus rospigliosii* [59], *Elateriospermum tapos* [146], *Microbiota decussata* [90], *Selaginella bryopteris* [106,141], *Selaginella moellendorffii* [142,143], *Taxus baccata* [127]
4	Sotetsuflavone	*Amentotaxus yunnanensis* [132], *Dacrydium araucarioides* [6], *Dacrydium pierrei* [147], *Selaginella denticulata* [108], *Selaginella tamariscina* [148], *Torreya yunnanensis* [132]
5	Ginkgetin	*Celaenodendron mexicanum* [45], *Cephalotaxus koreana* [47], *Chamaecyparis obtusa* [49], *Dacrydium araucarioides* [6], *Elateriospermum tapos* [146], *Selaginella doederleinii* [149], *Selaginella moellendorffii* [112,142,143], *Selaginella remotifolia* [115], *Selaginella stauntoniana* [121], *Taxus baccata* [127], *Taxus madia* [150], *Torreya nucifera* [131]
6	Isoginkgetin	*Chamaecyparis obtusa* [49], *Cycas circinalis* [56], *Gingko biloba* [137], *Podocarpus brevifolius* [144], *Podocarpus henkelii* [151], *Ranunculus ternatus* [99], *Selaginella doederleinii* [149]
7	Podocarpusflavone B	*Amanoa almerindae* [11], *Campylospermum calanthum* [38], *Celaenodendron mexicanum* [144], *Chamaecyparis obtusa* [49], *Decussocarpus rospigliosii* [59], *Elateriospermum tapos* [146], *Podocarpus brevifolius* [145]
8	4′,7′′-di-*O*-methylamentoflavone	*Cephalotaxus koreana* [47], *Selaginella remotifolia* [115], *Selaginella sinensis* [120], *Selaginella willdenowii* [124]
9	7,7′′-di-*O*-methylamentoflavone	*Amentotaxus yunnanensis* [132], *Chamaecyparis obtusa* [49], *Decussocarpus rospigliosii* [59], *Podocarpus imbricadus* [97], *Retrophyllum rospigliosii* [100], *Selaginella doederleinii* [109]
10	Heveaflavone	*Decussocarpus rospigliosii* [59], *Podocarpus imbricadus* [97], *Selaginella bryopteris* [106,138], *Selaginella doederleinii* [109], *Selaginella tamariscina* [148]
11	kayaflavone	*Ranunculus ternatus* [99], *Selaginella moellendorffii* [112]
12	Sciadopitysin	*Cephalotaxus fortunei* [46], *Cephalotaxus koreana* [47], *Cephalotaxus oliveri* [48], *Chamaecyparis obtusa* [49], *Cunninghamia lanceolata* [51], *Dacrydium araucarioides* [6], *Gingko biloba* [140], *Podocarpus brevifolius* [145], *Podocarpus nagi* [152], *Retrophyllum rospigliosii* [100], *Taxus baccata* [127], *Taxus madia* [150], *Torreya nucifera* [131], *Torreya yunnanensis* [132]
13	7,4′,7′′-tri-*O*-methylamentoflavone	*Retrophyllum rospigliosii* [100], *Taxus baccata* [153], *Taxus madia* [150]
14	7,4′,7′′,4′′′-tetra-*O*-methylamentoflavone	*Cephalotaxus koreana* [47], *Cephalotaxus fortunei* [46], *Dacrydium pierrei* [146], *Podocarpus brevifolius* [145], *Podocarpus henkelii* [151], *Podocarpus nagi* [152], *Retrophyllum rospigliosii* [100], *Selaginella denticulata* [108], *Selaginella doederleinii* [109,138,149], *Selaginella moellendorffii* [112], *Taxus baccata* [153], *Wollemia nobilis* [154]
15	7,4′,5′′,7′′,4′′′-penta-*O*-methylamentoflavone	*Cephalotaxus oliveri* [48]
16	3′′′-*O*-methylamentoflavone	*Lonicera macranthoides* [84]
17	6"-(2-hydroxy-3-methyl-3-butenyl)-amentoflavone	*Calophyllum venulosum* [37], *Garcinia bakeriana* [66]
18	6"-(3-methyl-2-butenyl)-amentoflavone	*Calophyllum venulosum* [37]
19	Garciniaflavone A	*Garcinia subelliptica* [73]
20	Garciniaflavone B	*Garcinia subelliptica* [73]
21	Garciniaflavone C	*Garcinia subelliptica* [73]
22	Garciniaflavone D	*Garcinia subelliptica* [73]
23	3′,8′′-biisokaempferide	*Nanuza plicata* [92]
24	5'- hydroxyamentoflavone	*Caesalpinia pyramidalis* [23]
25	Sumaflavone	*Selaginella tamariscina* [155,156]
26	Pyranoamentoflavone	*Calophyllum inophylloide* [30], *Calophyllum venulosum* [37]
27	7,4′-di-*O*-methylpyranoamentoflavone	*Calophyllum venulosum* [37]
28	7,4′′′-di-*O*-methylpyranoamentoflavone	*Calophyllum venulosum* [37]
29	Amentoflavone-7,4′,4′′′-tri-*O*-β-d-glucopyranoside	*Psilotum nudum* [157]
30	Amentoflavone-4′,4′′′-di-*O*-β-d-glucopyranoside	*Psilotum nudum* [157]
31	Amentoflavone-7,4′′′-di-*O*-β-d-glucopyranoside	*Psilotum nudum* [157]
32	Taiwanhomoflavone A	*Cephalotaxus wilsoniana* [158]

**Table 3:** Hydrogenation derivatives of amentoflavone.

No.	Compounds	Sources
33	(2*S*)-2,3-dihydro-7-*O*-β-d-glucopyranosylamentoflavone	*Cycas revoluta* [159]
34	(2*S*)-2,3-dihydro-7,7′′-di-*O*-β-d-glucopyranosylamentoflavone	*Cycas revoluta* [159]
35	(2′′*S*)-2′′,3′′-dihydro-4′-*O*-methylamentoflavone	*Selaginella remotifolia* [115], *Selaginella uncinata* [123,160]
36	(2*S*)-2,3-dihydro-4′-*O*-methylamentoflavone	*Cycas circinalis* [56], *Selaginella remotifolia* [115], *Selaginella uncinata* [123,137]
37	(2*S*,2′′*S*)-2,3,2′′,3′′-tetrahydro-4′-*O*-methylamentoflavone	*Cycas circinalis* [56]; *Selaginella uncinata* [123]
38	(2*S*,2′′*S*)-2,3,2′′,3′′-tetrahydroamentoflavone	*Cycas beddomei* [55,161], *Cycas revolute* [56], *Dysoxylum cauliflorum* [162], *Selaginella bryopteris* [106,141], *Selaginella uncinata* [123]
39	(2*S*)-2,3-dihydroamentoflavone	*Calophyllum venulosum* [37], *Cycas beddomei* [55,161], *Cycas pectinata* [58], *Cycas revoluta* [56], *Selaginella bryopteris* [106,141], *Selaginella mollendorfii* [142], *Selaginella remotifolia* [115], *Selaginella tamariscina* [163], *Selaginella uncinata* [123,137]
40	(2′′*S*)-2′′,3′′-dihydroamentoflavone	*Selaginella bryopteris* [106,141], *Selaginella remotifolia* [115], *Selaginella tamariscina* [163], *Selaginella uncinata* [123]
41	(2*S*,2′′*S*)-2,3,2′′,3′′-tetrahydroisoginkgetin	*Cycas circinalis* [56]
42	(2*S*)-2,3-dihydro-4′,4′′′-di-*O*-methylamentoflavone	*Cycas circinalis* [56]
43	(2*S*)-2,3-dihydro-4′′′-*O*-methylamentoflavone	*Selaginella remotifolia* [115]
44	(2*S*)-2,3-dihydro-7,7′′-di-*O*-methylamentoflavone	*Amentotaxus yunnanensis* [132]
45	(2*S*)-2,3-dihydro-4′′′-*O*-methylamentoflavone	*Cycas beddomei* [55,161]

**Table 4:** Other pharmacological effects of amentoflavone.

Function	Inducer	Model	Efficacy Evaluation	Reference
Anti-hyperlipidemia	High-cholesterol diet	Male Kunming mice	Decreased TG, TC, LDL-C in serum Increased HDL-C	[184]
Anti-hypertrophic scar	-	HSFBs	Inhibited cell viability, induced apoptosis Regulated Bax, TCTP, caspase-3, caspase-8, caspase-9	[185]
	-	SVECs	Inhibited cell viability Inhibited migration, invasion, tubular structure formation	
Anti-psoriasis	Imiquimod	Male BALBc Mice	Reduced skinfold thickening Improved erythema and scaling scores, histological lesions Suppressed increases of TNF-α, IL-17A, IL-22, IL-23	[186]
	M5 cocktail *	Human keratinocytes	Inhibited cell proliferation, promoted apoptosis Decreased overexpression of cyclin D1, cyclin E, IL-17A, IL-22 Inhibited the up-regulation of p65 NF-κB	
Anti-ulcerative colitis	Acetic acid	Male Wistar rats	Decreased mucosal injury score, vascular permeability Diminished LDH and MPO activity Increased GSH, SOD; decreased LPO, NO Reduced the colonic TNF-α, IL-1β, IL-6 Inhibited expression of iNOS and COX-2 Inhibited activation and translocation of NF-κB (p65/p50)	[187]
Hepatoprotection	CCl_4_	Male Kunming mice	Decreased GOT, GPT, hepatic MDA Increased hepatic SOD	[184]
Osteogenesis effect	-	Human mesenchymal stem cells	Enhanced proliferation, ALP activity, mineralization Upregulated expression of RUNX2, osterix proteins Increased the levels of phosphorylated JNK and p-p38	[188]
Radioprotection	Co-60 irradiation	V79 Chinese hamster lung fibroblast cells	Inhibited apoptosis, promoted the G2 phase Decreased the concentration of ROS and mitochondrial mass	[189]

ALP: alkaline phosphatase; COX-2: cyclooxygenase-2; GOT: glutamic oxaloacetic transaminase; GPT: glutamic pyruvic transaminase; HDL-C: high-density lipoprotein cholesterol; HSFBs: hypertrophic scar fibroblasts; iNOS: inducible nitric oxide synthase; LDH: lactate dehydrogenase; LDL-C: low-density lipoprotein cholesterol; RUNX2:runt-related transcription factor 2; SVECs: Simian virus-40-transformed murine endothelial cells; TC: total cholesterol; TCTP: translationally controlled tumour protein; TG: triglyceride; -: no inducer; *: IL-1α, IL-17A, IL-22, Oncostatin M, and TNF-α, each at 10 ng/mL for two days.
